# The role of hydraulic and geomorphic complexity in predicting invasive carp spawning potential: St. Croix River, Minnesota and Wisconsin, United States

**DOI:** 10.1371/journal.pone.0263052

**Published:** 2022-02-03

**Authors:** Alan Kasprak, P. Ryan Jackson, Evan M. Lindroth, J. William Lund, Jeffrey R. Ziegeweid

**Affiliations:** 1 U.S. Geological Survey, Central Midwest Water Science Center, Iowa City, IA, United States of America; 2 U.S. Geological Survey, Central Midwest Water Science Center, Urbana, IL, United States of America; 3 U.S. Geological Survey, Upper Midwest Water Science Center, Mounds View, MN, United States of America; Centro de Investigacion Cientifica y de Educacion Superior de Ensenada, MEXICO

## Abstract

Since they were first introduced to the United States more than 50 years ago, invasive carp have rapidly colonized rivers of the Mississippi River Basin, with detrimental effects on native aquatic species. Their continued range expansion, and potential for subsequent invasion of the Great Lakes, has led to increased concern for the susceptibility of as-yet uncompromised lotic and lentic systems in the central United States. Because invasive carp eggs and larvae must drift in the river current for the first several days following spawning, numerical drift modeling has emerged as a useful technique for determining whether certain river systems and reaches have the potential to support suspension-to-hatching survival of invasive carp eggs, a critical first step in recruitment. Here we use one such numerical modeling approach, the Fluvial Egg Drift Simulator (FluEgg), to estimate bighead carp (*Hypophthalmichthys nobilis*) egg hatching success and larval retention in a 47.8-kilometer (km) reach of the multi-thread St. Croix River, Minnesota and Wisconsin, United States. We explore three approaches for obtaining the hydraulic data required by FluEgg, parameterizing the model with either (a) field hydraulic data collected within the main channel during a high-flow event, or hydraulic data output from a one-dimensional hydrodynamic model with both (b) steady, and (c) unsteady flows. We find that the three approaches, along with the range of water temperatures and discharge used in simulations, produce vastly different predictions of streamwise transport and in-river egg hatching probability (0% for field data, 0 to 96% for steady-state hydraulic modeling, and 1.8 to 65% for unsteady modeling). However, all FluEgg simulations, regardless of the source of hydraulic data, predicted that no larvae reach the gas bladder inflation stage within the study reach where nursery habitat is abundant. Overall, these results indicate that the lower St. Croix River is suitable for invasive carp spawning and egg suspension until hatching for a range of discharge and water temperatures. These results highlight the role of complex channel hydraulics and morphology, particularly multi-thread reaches, and their inclusion in ecohydraulic-suitability modeling to determine susceptibility of river systems for invasive carp reproduction. Our work also emphasizes the scientific value of multi-dimensional hydrodynamic models that can capture the spatial heterogeneity of flow fields in geomorphically complex rivers. This work may help to guide management efforts based on the targeted monitoring and control and improve invasive carp egg and larvae sampling efficiency.

## 1. Introduction

Following their introduction to the southeastern United States in the 1960s and 1970s as biological control agents for aquaculture ponds, invasive carp have rapidly expanded to both lentic and lotic waterbodies throughout thef country, with particular emphasis on the Mississippi River Basin [1, 2]. Collectively, the general term ‘invasive carp’ encompasses four species: grass carp (*Ctenopharyngodon idella*), silver carp (*Hypophthalmichthys molitrix*), bighead carp (*H*. *nobilis*), and black carp (*Mylopharyngodon piceus*). At present, primarily bighead and silver carp have been captured throughout the Upper Mississippi, Ohio, and Illinois River Basins [2, 3], and there is concern that populations could become established in the Great Lakes [4, 5].

The detrimental effects of invasive carp on native aquatic ecosystems have been widely documented and can be subdivided into direct and indirect effects on native biota [6, 7]. Direct effects include competition with other species for food, which may include aquatic vegetation, plankton, and mollusks such as mussels and snails [8]. Indirect effects include carp removing aquatic vegetation and thus negatively affecting other fish species that that rely on aquatic vegetation for shelter or reproductive habitat [8]. Widespread disturbance of river and lake bottom substrates by feeding carp also reduces water quality by increasing turbidity from bed disturbance, with additional deleterious effects on aquatic organisms that depend on clear water [9, 10].

In riverine systems, recent efforts aimed at the detection and control of invasive carp populations have centered on monitoring through direct capture or chemical detection (e.g., environmental deoxyribonucleic acid (eDNA; [8, 11, 12]) of the locations of individual fish or populations in waterbodies. At the same time, research has focused on understanding the biophysical factors that lead to successful invasive carp spawning, and this work has developed predictive tools for forecasting where, and with what degree of success, these fish are likely to spawn in river systems [13]. Such predictive efforts have the potential to inform and refine targeted field sampling and subsequent removal of invasive carp [14].

Invasive carp typically spawn in the spring and summer months [13] during high streamflow events. Spawning locations are generally co-located with areas of hydraulic heterogeneity, including in the lee of sandbars and islands, near hardpoints such as rock outcroppings, in the turbulent tailwater of dams [14], and at channel confluences. Eggs are semibouyant and must remain in suspension (i.e., not settle onto the streambed) to remain viable until the time they hatch. Hatching time is largely a function of water temperature, with increasing water temperatures leading to more rapid development and earlier hatching after fertilization ([15]; Table 1). Immediately after hatching, larvae begin to swim vertically and are no longer at risk of settling and burial on the substrate [16]. Once the larval fish mature to the gas bladder inflation stage, they are able to swim laterally and begin to search for shallow, low-velocity nursery habitat [17]. The time required to reach this stage varies as a function of stream temperature and species, with higher temperatures leading to more rapid gas bladder inflation (Table 1). For invasive carps, gas bladder inflation occurred 125–135 hours following egg fertilization at a water temperature of 22° Celsius (C) in laboratory studies performed by George and Chapman [18, 19].

**Table 1:** FluEgg simulations performed for hypothetical bighead carp spawning in the St. Croix River (Minnesota, Wisconsin). Spawning is assumed to occur in the tailwater of the St. Croix Falls hydropower plant in the center of the channel and at the water surface for all simulations. All simulations released 10,000 bighead carp eggs at the spawning location and were run until larvae reached the gas bladder inflation stage (simulation time; computed from [18]). Hatching times are derived from [20].

Simulation number	Hydraulic data input type	Water temperature, in degrees Celsius	Discharge, in cubic meters per second	Simulation time, in hours	Spawning time (Central Daylight Time)	Hatching time, in hours (after spawning)
1	ADCP	18	variable	252.4	--	57.8
2	ADCP	19	variable	207.3	--	51.3
3	ADCP	20	variable	175.9	--	45.8
4	ADCP	21	variable	152.8	--	41.1
5	ADCP	22	variable	135.0	--	37.0
6	ADCP	23	variable	120.9	--	33.5
7	ADCP	24	variable	109.5	--	30.5
8	ADCP	25	variable	100.1	--	27.9
9	ADCP	26	variable	92.1	--	25.5
10	ADCP	27	variable	85.4	--	23.5
11	ADCP	28	variable	79.5	--	21.7
12	Steady HEC-RAS	18	283	252.4	--	57.8
13	Steady HEC-RAS	19	283	207.3	--	51.3
14	Steady HEC-RAS	20	283	175.9	--	45.8
15	Steady HEC-RAS	21	283	152.8	--	41.1
16	Steady HEC-RAS	22	283	135.0	--	37.0
17	Steady HEC-RAS	23	283	120.9	--	33.5
18	Steady HEC-RAS	24	283	109.5	--	30.5
19	Steady HEC-RAS	25	283	100.1	--	27.9
20	Steady HEC-RAS	26	283	92.1	--	25.5
21	Steady HEC-RAS	27	283	85.4	--	23.5
22	Steady HEC-RAS	28	283	79.5	--	21.7
23	Steady HEC-RAS	18	566	252.4	--	57.8
24	Steady HEC-RAS	19	566	207.3	--	51.3
25	Steady HEC-RAS	20	566	175.9	--	45.8
26	Steady HEC-RAS	21	566	152.8	--	41.1
27	Steady HEC-RAS	22	566	135.0	--	37.0
28	Steady HEC-RAS	23	566	120.9	--	33.5
29	Steady HEC-RAS	24	566	109.5	--	30.5
30	Steady HEC-RAS	25	566	100.1	--	27.9
31	Steady HEC-RAS	26	566	92.1	--	25.5
32	Steady HEC-RAS	27	566	85.4	--	23.5
33	Steady HEC-RAS	28	566	79.5	--	21.7
34	Steady HEC-RAS	18	991	252.4	--	57.8
35	Steady HEC-RAS	19	991	207.3	--	51.3
36	Steady HEC-RAS	20	991	175.9	--	45.8
37	Steady HEC-RAS	21	991	152.8	--	41.1
38	Steady HEC-RAS	22	991	135.0	--	37.0
39	Steady HEC-RAS	23	991	120.9	--	33.5
40	Steady HEC-RAS	24	991	109.5	--	30.5
41	Steady HEC-RAS	25	991	100.1	--	27.9
42	Steady HEC-RAS	26	991	92.1	--	25.5
43	Steady HEC-RAS	27	991	85.4	--	23.5
44	Steady HEC-RAS	28	991	79.5	--	21.7
45	Unsteady HEC-RAS	22	variable	135.0	06/19/2018 00:00	37.0
46	Unsteady HEC-RAS	22	variable	135.0	06/19/2018 08:00	37.0
47	Unsteady HEC-RAS	22	variable	135.0	06/19/2018 16:00	37.0
48	Unsteady HEC-RAS	22	variable	135.0	06/20/2018 00:00	37.0
49	Unsteady HEC-RAS	22	variable	135.0	06/20/2018 08:00	37.0
50	Unsteady HEC-RAS	22	variable	135.0	06/20/2018 16:00	37.0
51	Unsteady HEC-RAS	22	variable	135.0	06/21/2018 00:00	37.0
52	Unsteady HEC-RAS	22	variable	135.0	06/21/2018 08:00	37.0
53	Unsteady HEC-RAS	22	variable	135.0	06/21/2018 16:00	37.0
54	Unsteady HEC-RAS	22	variable	135.0	06/22/2018 00:00	37.0
55	Unsteady HEC-RAS	22	variable	135.0	06/22/2018 08:00	37.0
56	Unsteady HEC-RAS	22	variable	135.0	06/22/2018 16:00	37.0

Notation: ADCP, acoustic Doppler current profiler; --, not applicable; HEC-RAS, U.S. Army Corps of Engineers Hydrologic Engineering Center’s River Analysis System.

Direct field monitoring of the presence of invasive carp, along with their eggs and larvae, is invaluable for documenting successful spawning events. However, such campaigns can be financially and logistically intensive, and may omit river segments that cannot be sampled due to a variety of constraints [21]. As a potential alternative, predictive numerical modeling of spawning success based on reach-scale physical variables (e.g., river hydraulics and water temperature) has subsequently emerged as a tool for understanding river reaches where invasive carp may successfully reproduce. These techniques can help guide field sampling efforts [14] and increase the chance of successfully capturing fish at the reproductive life stage. Alternatively, numerical models can be used as hindcasts to pinpoint the most likely spawning sites when eggs or larval fish are captured at downstream locations [14].

One such numerical drift model is the Fluvial Egg Drift Simulator (FluEgg), which was developed by [13] and subsequently used in numerous studies of invasive carp spawning suitability on rivers across the Midwest and Great Lakes regions [20, 22–26]. FluEgg functions as a bio-physical particle tracking algorithm [14] that simulates the streamwise and vertical position of each simulated egg at user-defined timesteps. Egg positions at any model timestep are determined as a function of their relative settling velocity in the water column versus the hydraulic components of the flow acting to keep eggs in suspension. The model uses hydraulic data including flow depth, flow velocity, shear velocity, water temperature, and study reach length to estimate whether eggs will remain in suspension for a sufficient time that allows development to hatching. Ultimately, FluEgg provides an estimate of potential spawning success, expressed as a percentage of total simulated eggs at risk of hatching within the modeled reach, and estimates the streamwise distributions of eggs and larvae from spawning to the gas bladder inflation stage. The model can be parameterized with hydraulic data obtained via field surveys, or by using the output of steady or unsteady hydrodynamic models.

Natural rivers span a range of planforms, and produce a diverse suite of hydraulic characteristics that may vary appreciably over short distances on any individual waterway [27–29]. As a result, the input hydraulic data to FluEgg is of fundamental importance in the subsequent computation of invasive carp spawning suitability. As a simple illustrative example without diffusion (advection only), over the course of the 130 hours typically required for gas bladder inflation following spawning, an increase of 0.1 m/s in mean flow velocity can result in an additional 47 km distance traveled by drifting eggs and larvae. Such a difference can have an appreciable effect not only on whether eggs remain in suspension, but also can determine where eggs hatch in the river and where larvae begin searching for nursery habitat.

Field surveys can provide detailed information on river hydraulics, but they are typically limited in extent as a result of crew time, safety, and financial resources, and may only cover the main channel in multi-thread (i.e., braided or anastomosing) rivers. Alternatively, FluEgg inputs that are derived from numerical hydrodynamic models can predict flow conditions across wide swaths of the river valley and over long reaches. However, such models require detailed input data, a requisite level of investigator expertise in numerical modeling, and may introduce considerable uncertainty in their predictions at locations where field data are not co-located with model outputs for validation [30, 31]. To date, there has been little progress in understanding how each of these input data schemes (e.g., field data versus hydrodynamic model predictions) may influence invasive carp spawning success predictions derived from FluEgg. This knowledge gap has implications for our ability to predict where and when invasive carp may spawn successfully, and for the ability of natural resource managers and scientists alike to lead targeted detection and/or control efforts with confidence that resources are deployed in the right place and time for optimal success.

Here we evaluate three methods for quantifying potential invasive carp spawning success on a geomorphically and hydraulically diverse river system. Our goal is to determine whether and how input data source affects resultant spawning suitability predictions. Specifically, we complete spawning suitability modeling for bighead carp on a 47.8-km reach of the multi-threaded St. Croix River, Minnesota and Wisconsin, United States. Since 2015, several mature bighead carp (male and female) and one silver carp have been caught in the lower St. Croix River; however, spawning activity has not been detected within this reach as of December 2020 [32]. We use input hydraulic data derived from (a) field-based surveys, which were limited to capturing main-channel hydraulics and geometry, and (b) a one-dimensional (1D) hydraulic model with both steady and unsteady streamflow, developed using airborne lidar and bathymetric surveys that captured the main channel and numerous anabranches throughout the study reach. We then compare the results of these three approaches and their respective assessments of bighead carp spawning potential along the study reach. Our results have implications for planning and conducting field surveys and numerical modeling studies aimed at forecasting the potential for invasive carp spawning and recruitment across a wide range of river types.

## 2. Study setting

### 2.1. Physical characteristics

**Fig 1:**
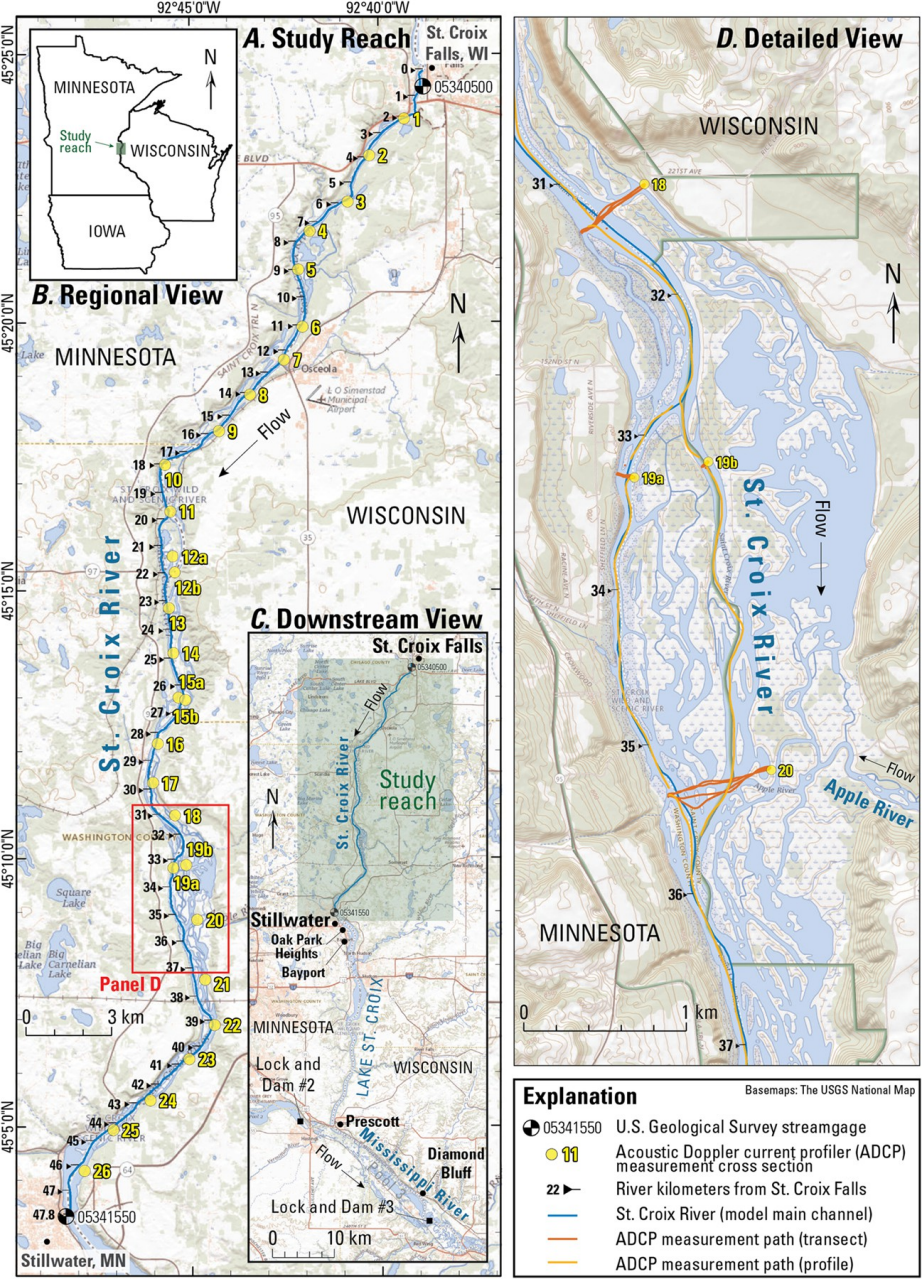
Study area overview map of the 47.8-kilometer St. Croix River study reach between St. Croix Falls, Wisconsin, and Stillwater, Minnesota. (A) acoustic Doppler current profiler (ADCP) data collected during June 19–22, 2018, where yellow dots show locations of transect stations; (B) regional overview inset map; (C) downstream map showing the study reach in the context of the lower St. Croix River to its confluence with the Mississippi River; (D) detailed inset image depicting ADCP data collection in a multi-thread segment of the study reach.

The St. Croix River flows generally south for approximately 270 km from northwestern Wisconsin, USA to its confluence with the Mississippi River near Prescott, Wisconsin. The study reach examined here is the 47.8-km segment between St. Croix Falls, Wisconsin, and Stillwater, Minnesota. Within this reach, the river forms the state boundary between Wisconsin and Minnesota (Fig 1). Approximately the upstream 6 km of the study reach are underlain by basalts and tuffs of the Mesoproterozoic Clam Falls volcanic sequence [33], and these resistant lithologies give rise to a confined river planform within a narrow bedrock gorge. Over the remainder of the study area, the bedrock geology alternates between sequences of Paleozoic sandstones and shales [33]. Throughout this reach, the river planform alternates between a single-threaded channel and segments exhibiting a braided planform, where flow is split into two or more anabranches; these areas are marked by vegetated islands and bare sandbars. The hydro-geomorphic characteristics of the St. Croix, including the presence of bedrock hardpoints and tributary/anabranch confluences, are similar to rivers in the native range of bighead carp [34], as well as other systems in the U.S. that have undergone colonization. The St. Croix River is protected under the National Wild and Scenic Rivers Act of 1968, and there is an approximately 1-km wide forested riparian buffer area on each bank of the river, with the exception of the reach adjacent to Stillwater where development encroaches up to the river course.

Discharge throughout the study reach is affected by a hydroelectric power dam at St. Croix Falls; however, since 2006 this facility has been operated so as to have minimal effects on downstream water levels, as outlined in a 2006 memorandum of understanding between the dam operator, Xcel Energy, and the Wisconsin Department of Natural Resources [35]. Beginning near Stillwater, river stage is affected by the backwater resulting from impoundment of the main stem Mississippi River (to which the St. Croix is a tributary) for navigation at the U.S. Army Corps of Engineers’ Lock and Dam #3 near Diamond Bluff, Wisconsin (Fig 1). At the U.S. Geological Survey (USGS) St. Croix Falls streamgage (05340500; Fig 1), the mean annual flow between October 1, 1910, and September 30, 2015, is approximately 125 cubic meters per second (m^3^/s; [36]). Analysis of discharge records between 1902 and 2000 indicate the discharges for the 50 percent annual exceedance probability (AEP) and the 20 percent AEP floods are 631 m^3^/s and 929 m^3^/s, respectively [37].

### 2.2. Status of invasive carp invasion in the study reach

Since 1991, individual captures of grass, bighead, and silver carp have occurred in several rivers and lakes within Minnesota and Wisconsin, including the backwater reach of the St. Croix River downstream of Stillwater and pool 3 of the Mississippi River. However, monitoring efforts to date have not detected early life stages in these reaches [32]. In May of 2018, two bighead carp were captured by the Minnesota Department of Natural Resources approximately 6 km downstream of Stillwater, near Bayport, Minnesota ([32]; Fig 1); while both fish were mature and capable of reproduction, neither showed indications of recent spawning activity. More recently in 2019, a bighead carp was captured in the St. Croix just downstream from Stillwater, near Oak Park Heights, Minnesota, and a silver carp was caught by a commercial fisherman in the St. Croix near Prescott, Wisconsin. The presence of invasive carp in the St. Croix River, and documented evidence of reproduction in other geomorphically similar waterways around the Great Lakes region [38] has led to concern that invasive carp may reproduce in the lower St. Croix River.

## 3. Methods

We simulated the potential for successful spawning of bighead carp in the St. Croix River between St. Croix Falls, Wisconsin, and Stillwater, Minnesota, using FluEgg [39], driving the drift model with three hydraulic datasets. The first of these datasets consisted of hydraulic and bathymetric data collected in the field using an acoustic Doppler current profiler (ADCP), while the second and third cases used output from a steady and unsteady 1D hydrodynamic model, respectively. The hydraulic model was developed by the U.S. Army Corps of Engineers (USACE; [40]) and was updated for the present study to include bathymetric channel data collected via the ADCP field surveys and topographic data from remote sensing. Below we discuss each of these three approaches in detail, followed by a description of the FluEgg model and its operation.

### 3.1. Field data collection and processing

**Fig 2:**
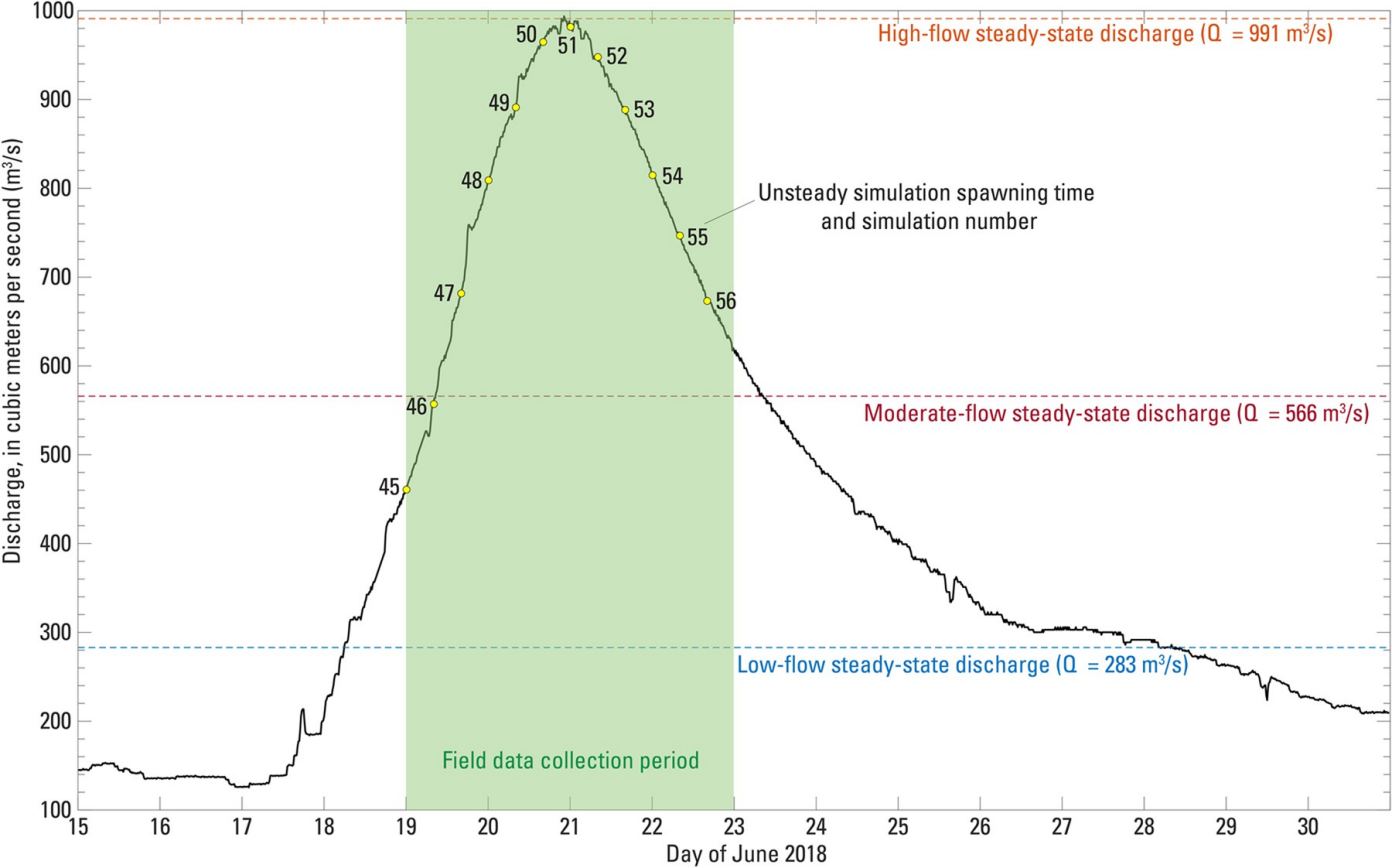
St. Croix River 15-minute discharge record in June 2018, as measured at St. Croix Falls, Wisconsin, (U.S. Geological Survey streamgage 05340500; [42]). The field data collection period (green patch), discharge values used for steady-state hydraulic model simulations (dashed lines), and spawning times for unsteady simulations (yellow dots) are also shown for reference.

Field data describing channel hydraulics, bathymetry, and water temperature were collected during the 4-day period spanning June 19–22, 2018, along the 47.8-km reach between St. Croix Falls and Stillwater (Fig 1; [37]). These data were collected by a USGS survey crew using a manned boat equipped with a Teledyne RDI RiverRay ADCP and YSI multiparameter water-quality sonde coupled with a Trimble R10 real-time kinematic global positioning system (RTK-GPS) unit. During field data collection, discharge at the St. Croix Falls USGS streamgage (05340500) varied between 462 m^3^/s and 994 m^3^/s ([1]; Fig 2). The peak discharge of 994 m^3^/s approximately corresponds to the 20-percent AEP flood discharge at the St. Croix Falls streamgage. ADCP and water-quality data were collected as a combination of cross sections oriented perpendicular to the flow direction and as streamwise profiles collected during travel between cross-section locations. The mean cross-section spacing was 1.8 km and a total of four transects were collected at each cross section along with a 5-minute (min) stationary (at-a-point) measurement of velocity near the thalweg of each cross section; these stationary measurements were used to determine whether bed sediment was actively being transported (i.e., a moving-bed test) and to aid in post-processing of ADCP data.

**Fig 3:**
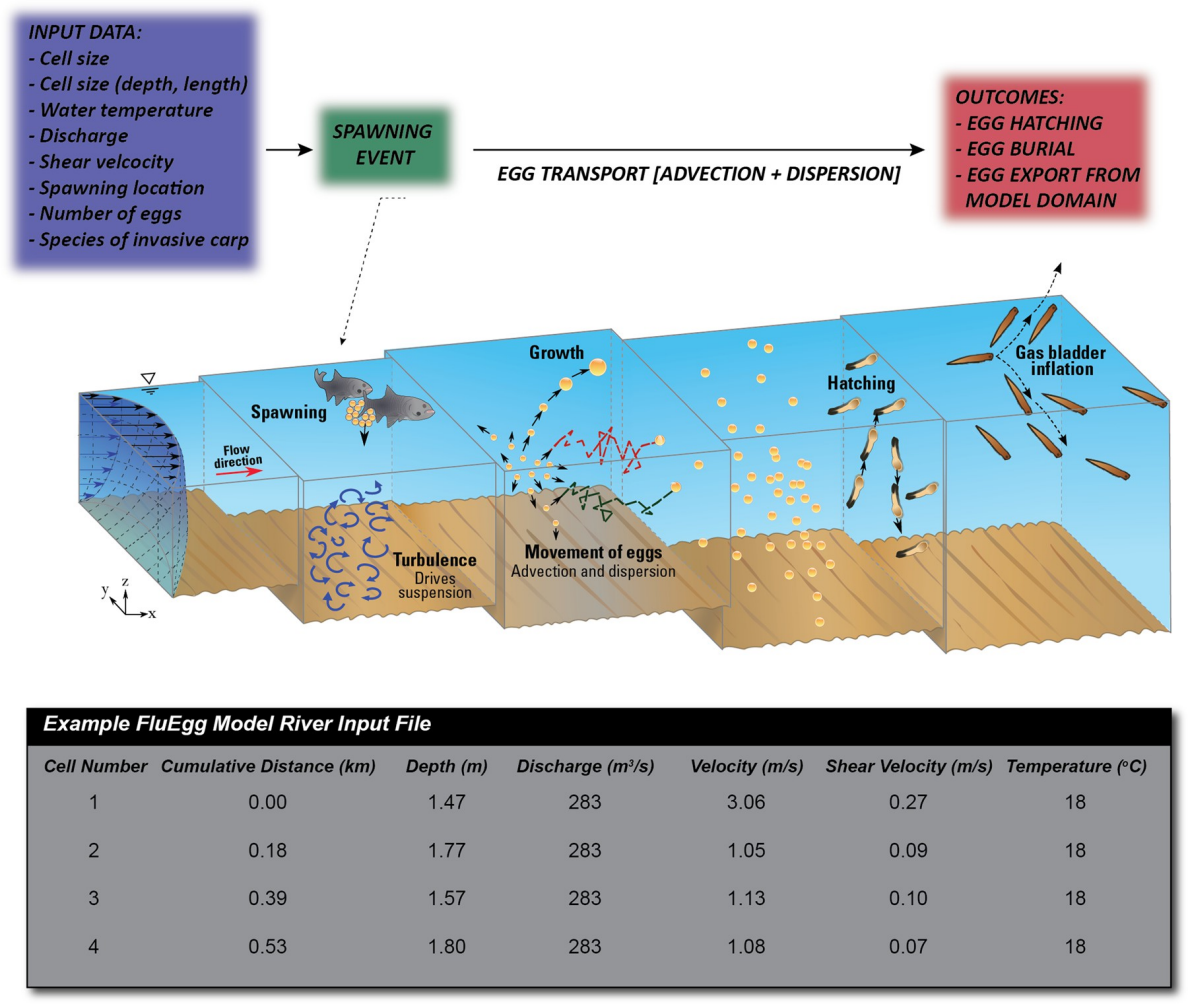
FluEgg model operation schematic depicting input data and model workflow. Bottom panel shows an example of FluEgg river input text file that provides information on reach dimensions, hydraulics, and water temperature for simulations. Figure republished from [13] under a CC BY license, with permission from Elsevier, original copyright 2013.

ADCP data were post processed using WinRiverII Version 2.18, and ASCII text files were exported for use in the Velocity Mapping Toolbox software (VMT, Version 4.09; [41]). Using VMT, georeferenced ADCP data were further processed to produce bathymetry data for model development, 2-min time-averaged velocity data along the main channel (Fig 1A, white line), and 3-dimensional mean velocity fields and estimates of shear velocity at each cross section in Fig 1A. These data were used to build river input files for FluEgg modeling (see Section 3.3 and Fig 3 for a description of these parameters). Mean water temperature at each transect was obtained using YSI sonde data collected concurrently with ADCP surveys. Additional water-quality properties including specific conductance, pH, and dissolved oxygen were not used in this study, but are reported in the associated USGS data release [43].

Field data were largely constrained to the main channel, where sufficient depths and a lack of obstructions allowed collection of requisite hydrodynamic data (Fig 1). Sampling constraints arising from safety concerns related to boat-based data collection in a braided river during high flow conditions prevented additional sampling of side channels and backwater areas. Furthermore, the large extent of the study reach minimized the time that could be spent sampling side channels and backwaters. In some instances (e.g., Transects 12, 15, and 19), ADCP data were collected within multiple anabranches, but in general, wider areas of the river were left largely unsurveyed via ADCP.

### 3.2. Hydraulic modeling

As an alternative to driving FluEgg simulations using field-collected ADCP data (Section 3.1. above), we used a 1D hydraulic model to derive input data for subsequent FluEgg modeling. We used the USACE Hydrologic Engineering Center’s River Analysis System software (HEC-RAS; Version 5.0.6). HEC-RAS has been extensively reviewed and used in the literature [44–46]. When run in 1D mode, as was the case here, the model uses input channel bathymetry and upland topography, along with boundary conditions that typically consist of river discharge at the upstream model domain boundary and flow stage at the downstream boundary. For steady (i.e., time-invariant) discharge simulations, HEC-RAS iteratively solves the 1D energy equation [47]. For unsteady (i.e., time-varying) discharge applications, the model solves the 1D Saint Venant equations using finite differencing [47]. In both cases, hydraulic parameters (e.g., flow depth, velocity, shear stress) are computed as model outputs at user-defined cross sections spaced throughout the reach of interest.

The HEC-RAS model used here was generated by the USACE Corps Water Management System from an original model iteration developed for the St. Croix River by the U.S. National Oceanic and Atmospheric Administration’s North Central River Forecast Center [40]. This model is part of a broader effort to develop hydraulic models for the Upper Mississippi River Basin. Channel bathymetry was developed using both the ADCP-surveyed cross sections described above, along with known individual USACE-surveyed cross sections, using linear interpolation in the HEC-RAS geometry editor. Topographic data for the remainder of the river valley were obtained from 1-meter (m) resolution airborne light detection and ranging (i.e., lidar) digital elevation models [48] and merged with bathymetric data to create a single continuous elevation grid. Mean cross-section spacing in the HEC-RAS model was 0.28 km. The upstream-most cross section (and hence the upstream model domain boundary) was located immediately below the dam at St. Croix Falls, 500 m upstream from USGS streamgage 05340500, and the downstream model boundary was co-located with the USGS streamgage at Stillwater, Minnesota (05341550; Fig 1). We assumed that there was negligible discharge or stage change in the 500-m reach between the upstream model boundary and the St. Croix Falls streamgage.

The HEC-RAS model was run for two scenarios, (a) steady flow and (b) unsteady flow. For steady flow simulations, three constant discharges were specified: 283 m^3^/s, 566 m^3^/s, and 991 m^3^/s, reflective of low, moderate, and high discharges during the field sampling period in June 2018 (Fig 2). Corresponding downstream stage for each of these discharges was extracted from the record at the USGS Stillwater streamgage [49] and used as a downstream model boundary condition. For unsteady flow modeling, the 15-minute records of discharge at the USGS St. Croix Falls streamgage and stage at the USGS Stillwater streamgage were used as upstream and downstream model boundary conditions, respectively, and the model was run for the period between June 15 and June 30, 2018. Model outputs were produced at 15-minute intervals throughout this period. We adjusted model roughness (specified as a Manning’s *n* value in HEC-RAS) until simulated water surface elevations at individual cross sections best matched the corresponding field-observed values surveyed during the June 19–22 field sampling effort. Adjustment of roughness values was completed when the mean water surface elevation error, defined as the difference between simulated and observed water surface elevations, was minimized and showed no appreciable change when roughness values were further changed. The same roughness values obtained through this calibration procedure were subsequently used for steady flow simulations.

From each steady flow simulation, we exported a text file containing (1) locations of each cross section, (2) discharge, (3) mean cross-section flow depth, (4) mean cross-section flow velocity, and (5) mean cross-section shear velocity from HEC-RAS for use in subsequent FluEgg spawning suitability simulations. For the unsteady flow simulation, the required hydraulic data are extracted at each timestep from the HEC-RAS model output by FluEgg directly.

### 3.3. FluEgg modeling

We used the FluEgg drift model version 4.1 [39] to quantify the potential for successful hatching of bighead carp eggs. FluEgg uses hydraulic and channel geometry data obtained from either (a) field-based surveys (Section 3.1) or (b) numerical model outputs (Section 3.2). In addition to these data, FluEgg requires input data describing water temperature in the modeled reach, along with biological information including invasive carp species, the number of eggs to simulate, and the hypothetical location of spawning within the reach. In the present study, 10,000 bighead carp eggs were hypothetically spawned in the center of the channel and at the water surface immediately downstream from the dam at St. Croix Falls, Wisconsin. We used 10,000 eggs in each simulation because it struck an ideal balance between minimizing simulation run time and output file sizes while still allowing us to develop a clear picture of egg plume transport. The model simulates egg size and densities using distributions (i.e., mean and standard deviation) developed from laboratory observations [13]. The requisite hydraulic data for a FluEgg simulation was provided by a river input file for steady-flow simulations; an example of a portion of one such file is shown in Fig 3. In addition to the hydraulic data used in this study, we note that FluEgg can also incorporate the mean vertical and lateral components of flow velocity for each model cell [13]; however, these components were set to zero for all FluEgg simulations in the present study. This assumption is valid in the present study as continuity of mass requires these components to sum to zero for a reach (or cell) with no net change in discharge (flow can only enter and exit a cell in the streamwise direction) and all hydraulic modeling was 1D, and thus did not resolve flow velocity components other than those in the streamwise direction.

Here, we ran FluEgg simulations with a constant time step of 7 seconds (s); this was the minimum time step that met the FluEgg stability criteria for the parabolic-constant eddy diffusivity profile for all simulations [13]. Each simulation concluded when total simulation time reached the characteristic larval gas bladder inflation time of bighead carp for the simulated water temperature; this simulation time varied between 79.5 hours for a maximum simulated water temperature of 28°C and 252.4 hours for the minimum simulated temperature of 18°C (Table 1).

During the simulation, FluEgg tracks those eggs that settle to the channel bed (and would thus become non-viable because of a lack of oxygen and burial in bed sediment [50] versus those that are maintained in suspension throughout the simulation. FluEgg also tracks any eggs and larvae that are transported out of the model domain (i.e., past the downstream model boundary) during the simulation period. Full results files, including egg positions for all 10,000 eggs at every time step, were saved for each simulation. From these files, we extracted several parameters for subsequent analysis. These included (a) the positions of all eggs at the time of hatching, (b) the position of all larvae at the time of gas bladder inflation, and (c) the water temperature used in the simulation. Using these parameters, we computed the percentage of eggs at risk of hatching within the model domain and percentage of larvae that reach the gas bladder inflation stage within the model domain. The requirements of the former account for egg settling by assuming that eggs in the lower 5-percent of the water column at the time of hatching are not viable [13, 20].

## 4. Results

Here we present the results of 56 FluEgg simulations of hypothetical bighead carp spawning in the lower St. Croix River. We first detail the results of FluEgg simulations that used field-based hydraulic and water temperature data collected during June 19–22, 2018, between St. Croix Falls, Wisconsin, and Stillwater, Minnesota (Section 4.1). We then discuss the results of FluEgg simulations that used HEC-RAS hydraulic models to provide inputs to FluEgg (Section 4.2), beginning with a brief synopsis of model validation (Section 4.2.1) and then turning to FluEgg results from steady (i.e., constant discharge) hydraulic simulations (Section 4.2.2) and finally FluEgg results from unsteady (i.e., time-varying discharge) hydraulic simulations (Section 4.2.3). For all 56 simulations, all larvae were transported out of the study reach before reaching the gas bladder inflation stage. Therefore, the remainder of this section will focus on the assessment of the risk of egg hatching within the lower St. Croix River between St. Croix Falls, Wisconsin, and Stillwater, Minnesota.

### 4.1. FluEgg modeling using ADCP data

**Fig 4:**
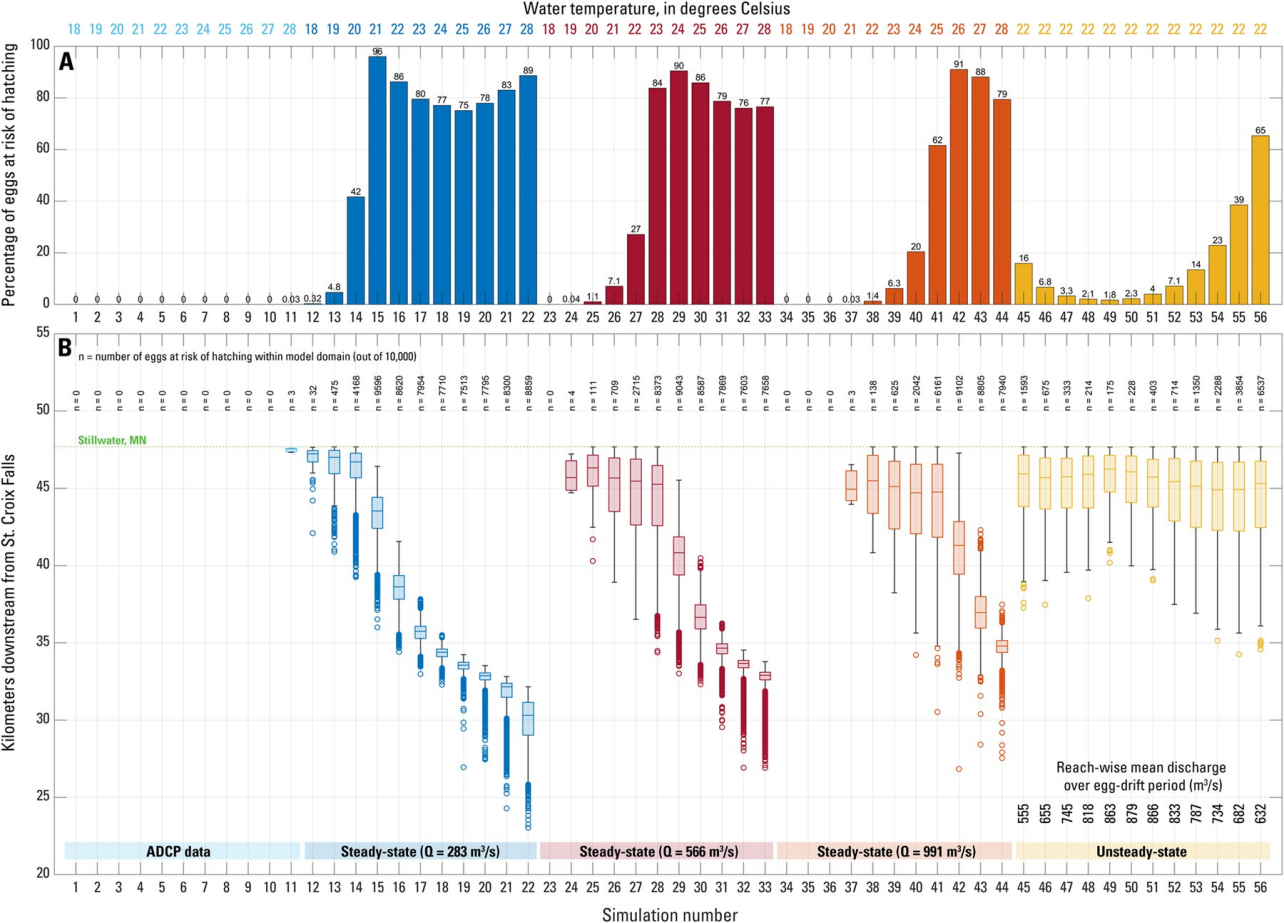
Results of the 56 FluEgg simulations listed in Table 1 for hypothetical bighead carp spawning on the lower St. Croix River. (A) Percentage of eggs spawned at St. Croix Falls, Wisconsin, at risk of hatching within the study reach. (B) Boxplots showing the streamwise distribution of the eggs at risk of hatching for each simulation at the time of egg hatching. The number of eggs at risk of hatching (n) is shown above each boxplot. The reach-wise mean discharge averaged over the egg drift period (spawning to hatching) for each unsteady simulation are shown for reference. No data are shown for eggs that have passed Stillwater, Minnesota, and left the model domain. The box and whiskers from bottom to top represent the nonoutlier minimum value, lower quartile, median value, upper quartile, and nonoutlier maximum value. Outliers are denoted by open circles.

We used hydraulic data from ADCP longitudinal profile and transect surveys (Section 3.1) to parametrize and run FluEgg simulations across a range of water temperatures from 18–28°C. For reference, the mean water temperature during June 2018 field surveys was 22°C. The probability of bighead carp eggs successfully hatching within the study reach is shown in Fig 4A. When driven with ADCP data primarily collected in the main channel, FluEgg predicts that simulated eggs were transported out of the study reach between St. Croix Falls, Wisconsin, and Stillwater, Minnesota, prior to egg hatching for all water temperatures simulated except 28°C (Fig 4A; Table 2). For all but the highest water temperature simulated, the entire egg plume was transported past Stillwater, Minnesota, and entered Lake St. Croix where the velocities decrease and there is a higher likelihood of egg settlement. At a water temperature of 28°C, only 3 eggs (0.03%) remained in the study reach (Fig 4B). The 3 remaining eggs are predicted to hatch less than 500 m upstream from Stillwater, Minnesota. When driven with ADCP data collected primarily from the main channel, FluEgg predicts that no larvae will reach the gas bladder inflation stage within the study reach for the range of water temperatures simulated.

**Table 2:** Tabular results of FluEgg simulations performed for hypothetical bighead carp spawning in the St. Croix River (Minnesota and Wisconsin). Spawning is assumed to occur in the tailwater of the St. Croix Falls hydropower plant (river kilometer 0.0) in the center of the channel and at the water surface for all simulations. All simulations released 10,000 bighead carp eggs at the spawning location and were run until larvae reached the gas bladder inflation stage.

Simulation number	Number of eggs downstream of study reach at hatching time	Number of eggs predicted to settle in study reach	Number of eggs at risk of hatching within study reach	Median position of eggs at risk of hatching within study reach (rkm)	Range of eggs at risk of hatching within study reach (km)	Number of larvae at risk of reaching gas bladder inflation within study reach
1	10000	0	0	--	--	0
2	10000	0	0	--	--	0
3	10000	0	0	--	--	0
4	10000	0	0	--	--	0
5	10000	0	0	--	--	0
6	10000	0	0	--	--	0
7	10000	0	0	--	--	0
8	10000	0	0	--	--	0
9	10000	0	0	--	--	0
10	10000	0	0	--	--	0
11	9997	0	3	47.5	0.3	0
12	9965	3	32	47.2	5.6	0
13	9500	25	475	47.0	6.8	0
14	5638	194	4168	46.7	8.4	0
15	0	404	9596	43.5	10.4	0
16	0	1380	8620	38.6	7.1	0
17	0	2046	7954	35.8	4.8	0
18	0	2290	7710	34.4	3.2	0
19	0	2487	7513	33.6	7.3	0
20	0	2205	7795	32.9	6.1	0
21	0	1700	8300	32.2	8.5	0
22	0	1141	8859	30.3	9.1	0
23	10000	0	0	--	--	0
24	9996	0	4	45.7	2.5	0
25	9882	7	111	46.3	7.4	0
26	9255	36	709	45.7	8.8	0
27	6894	391	2715	45.5	11.2	0
28	473	1154	8373	45.3	13.3	0
29	0	957	9043	40.8	12.5	0
30	0	1413	8587	36.6	8.2	0
31	0	2131	7869	34.7	6.7	0
32	0	2397	7603	33.7	7.6	0
33	0	2342	7658	32.9	6.9	0
34	10000	0	0	--	--	0
35	10000	0	0	--	--	0
36	10000	0	0	--	--	0
37	9995	2	3	44.9	2.6	0
38	9848	14	138	45.5	6.9	0
39	9307	68	625	45.1	9.5	0
40	7748	210	2042	44.7	13.5	0
41	3184	655	6161	44.8	17.2	0
42	0	898	9102	41.3	20.4	0
43	0	1195	8805	37.0	13.9	0
44	0	2060	7940	34.8	9.9	0
45	8218	189	1593	45.9	10.4	0
46	9234	91	675	45.7	10.2	0
47	9636	31	333	45.7	8.1	0
48	9765	21	214	45.9	9.8	0
49	9813	12	175	46.2	7.5	0
50	9730	42	228	46.1	7.7	0
51	9549	48	403	45.7	8.7	0
52	9177	109	714	45.4	10.2	0
53	8452	198	1350	45.1	10.8	0
54	7404	308	2288	44.9	12.5	0
55	5557	589	3854	44.9	13.4	0
56	2392	1071	6537	45.3	13.1	0

Notation: rkm, river kilometer measured downstream from St. Croix Falls (see Fig 1); km, kilometers; --, not applicable.

### 4.2. FluEgg modeling using hydraulic model output

We ran HEC-RAS simulations for the study reach under both steady (i.e., time-invariant) discharge and unsteady (i.e., time-varying) discharge scenarios. Here we describe results of model validation against field-collected data, along with the results of FluEgg simulations parameterized by both methods.

#### 4.2.1 Hydraulic model validation

**Fig 5:**
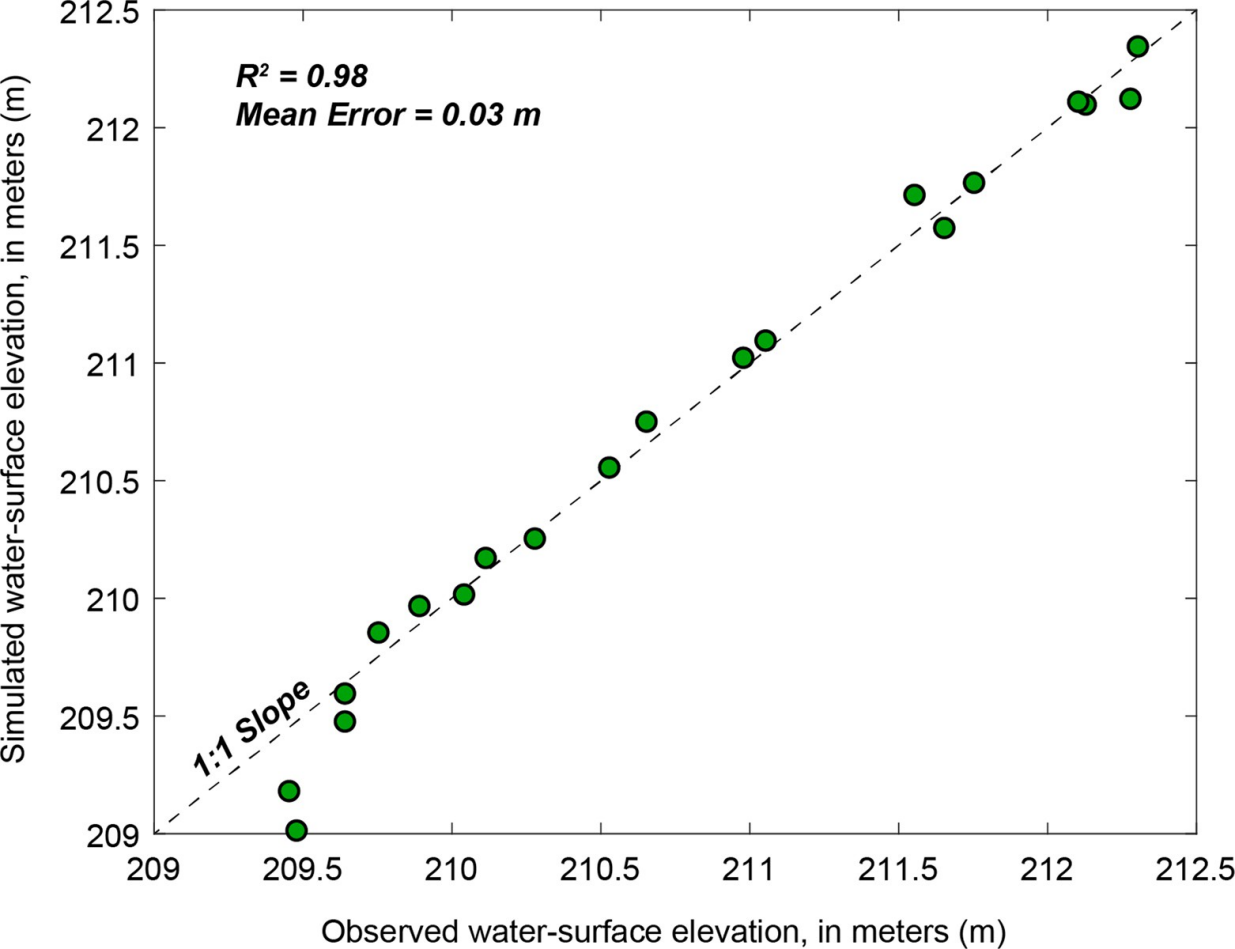
Field surveyed (observed) water surface elevation during June 2018 acoustic Doppler current profiler survey versus unsteady U.S. Army Corps of Engineers Hydrologic Engineering Center’s River Analysis System (HEC-RAS) simulated water surface elevation for corresponding date and time.

To verify the fidelity of HEC-RAS models, we compared simulated water surface elevation (WSE) against field-surveyed values at transects where RTK-GPS data were available. From unsteady HEC-RAS model outputs, we extracted the nearest simulated value of WSE in both time (i.e., the closest 15-minute resolution hydraulic output) and space (i.e., the nearest HEC-RAS transect to the relevant field observation). The locations of these validation points are shown in Fig 1A (yellow dots), and the relation between observed and simulated WSE is shown in Fig 5. We observed strong correlation between these two values (R^2^ = 0.98, mean error in WSE = 0.03 m; Fig 5). Because HEC-RAS modeling was completed across the entire range of discharges observed in the June 2018 survey period, this correlation indicates that the model performs well across the range of discharges during this period, which spanned from 462 m^3^/s to 994 m^3^/s at the USGS St. Croix Falls streamgage.

**Fig 6:**
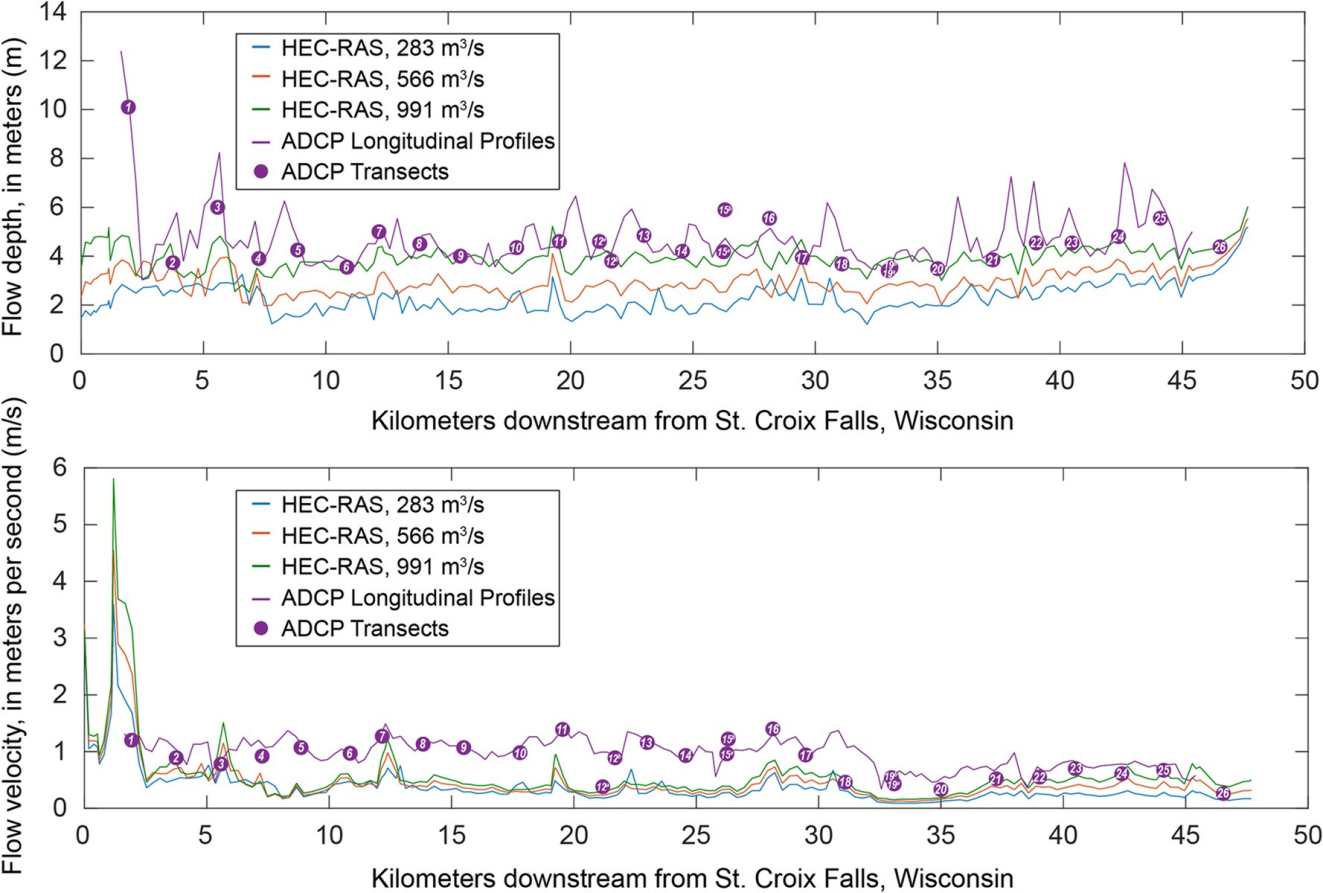
Plots of simulated U.S. Army Corps of Engineers Hydrologic Engineering Center’s River Analysis System (HEC-RAS) and observed (acoustic Doppler current profiler [ADCP]) flow depths throughout the 47.8-km study reach shown in the top panel. Bottom panel shows simulated and observed flow velocities for the same reach. Observed depths and velocities were collected during June 2018 ADCP surveys, while HEC-RAS results are obtained from steady-state simulations of three representative discharges during the June 2018 sampling period.

The greatest discrepancy between simulated and observed WSE was seen at the two downstream-most validation points (near transects 25 and 26; Fig 1A), where the mean WSE error was 0.37 m; that is, the field-surveyed WSE was higher than the HEC-RAS model prediction. In this portion of the study reach, backwater effects from Lake St. Croix caused by the impoundment of the Mississippi River downstream result in a flattening of the water surface at the downstream end of the study reach. As a result, the discrepancy between simulated and observed WSE, specifically the under prediction of WSE by the HEC-RAS model in this downstream portion of the reach is not surprising. For the remainder of the study area, the model performed well when evaluated against field observations. In general, the water depths and velocities predicted by HEC-RAS simulation follow similar longitudinal patterns as those observed during June 2018 survey, but the output of the techniques diverges at highly braided sub-reaches along the St. Croix River (Figs 1 and 6).

#### 4.2.2 Steady-state simulations

We ran time-invariant discharge HEC-RAS simulations at 283 m^3^/s, 566 m^3^/s, and 991 m^3^/s and used the resulting hydraulic data to drive FluEgg simulations in each case for a range of water temperatures from 18–28°C. Results of these simulations are shown in Fig 4. Unlike ADCP-driven FluEgg simulations, FluEgg simulations driven with output from the 1D steady-state hydraulic model predict egg hatching within the study reach (Fig 4A). In general, hatching rates decreased with higher discharge and lower water temperatures (Fig 4B). The mean (and range) of predicted hatch rates within the study area are 65% (0.3 to 96%), 48% (0 to 90%), and 32% (0 to 91%) for constant discharges of 283 m^3^/s, 566 m^3^/s, and 991 m^3^/s, respectively. Thus, all three discharges produced possible hatching rates of 90% or above. However, a higher water temperature was necessary as discharge increased—peak hatching rates occurred at 21°C, 24°C, and 26°C, for discharges of 283 m^3^/s, 566 m^3^/s, and 991 m^3^/s, respectively. The minimum water temperature required to produce in-reach egg hatching increased with increasing discharge. The percentage of eggs at risk of hatching only exceeded 10 percent for water temperatures above 19°C, 21°C, and 23°C, for discharges of 283 m^3^/s, 566 m^3^/s, and 991 m^3^/s, respectively.

As with the ADCP-data driven FluEgg simulations discussed above (Section 4.1), we computed the streamwise distribution of the egg plume using only those eggs that were predicted to remain in the study reach at the temperature-dependent hatching time and in the upper 95% of the water column (to account for settling). In general, higher temperatures and lower discharges resulted in eggs hatching farther upstream (Fig 4B). However, in cases where the egg plume was wholly within the study reach at the time of hatching (e.g., Fig 4B, simulations 15–22), egg settlement in wider, slower reaches (e.g., river kilometer [rkm] 32–36) resulted in up to 21 percent variability in predicted hatching rates (Fig 4A; Table 2).

As was the case with the ADCP-data driven FluEgg simulations, FluEgg simulations driven with output from the steady-state hydraulic model predicted that bighead carp larvae were already transported out of the study reach when they reached the gas bladder inflation stage (Table 2).

#### 4.2.3 Unsteady simulations

FluEgg simulations driven by output from the unsteady hydraulic model have a time-variant discharge; therefore, the spawning time must be specified, and results depend on when spawning occurred relative to the discharge hydrograph. Spawning was specified at 8-hour intervals beginning June 19, 2018, at 00:00 and ending June 22, 2018, at 16:00, for a total of 12 simulations (Table 1; Figs 2 and 4; simulations 45–56). We used a constant water temperature of 22°C for all unsteady simulations. FluEgg predicted between 1.8% and 65% of spawned eggs would be susceptible to hatching within the study reach (Fig 4A). The minimum predicted hatching rate occurs for simulation 49, in which spawning occurs about 16-h prior to the peak of the hydrograph at St. Croix Falls, Wisconsin, and the maximum predicted hatching rate occurs for simulation 56, the simulation with the latest spawning time on the falling limb of the hydrograph (Fig 2). In general, the predicted hatching rate is higher for spawning on the falling limb of the hydrograph (simulations 51–56) compared to spawning on the rising limb of the hydrograph (simulations 45–50) for equivalent discharge (Figs 2 and 4). This result, while dependent on the site and shape of the hydrograph, correlates well with some spawning patterns of bighead carp in nature where spawning was observed during and following peak discharge [51].

The centroid of the egg plumes remaining within the study reach at the 37-hour hatching time for unsteady simulations 45–56 exhibited relatively little variation, moving between rkm 44.9 and 46.2 (Figs 1 and 4B). However, the number of eggs making up these plumes and the overall extent of the plumes had substantially more variation. Unsteady simulations with the greatest number of eggs at risk of hatching also had the greatest plume extent as measured by the range (Fig 4B; Table 2). Except for simulation 56, the majority of the eggs spawned were transported downstream from Stillwater, Minnesota, prior to egg hatching, thus increasing the likelihood of egg settling in the low velocity environment of Lake St. Croix.

As was the case with the ADCP-data driven and steady-state FluEgg simulations, FluEgg simulations driven with output from the unsteady hydraulic model predicted no bighead carp larvae remained in the study reach between St. Croix Falls, Wisconsin, and Stillwater, Minnesota, when they reached the gas bladder inflation stage (Table 2).

## 5. Discussion

### 5.1. Suitability of the St. Croix River for invasive carp spawning

Results of FluEgg simulations using both steady and unsteady 1D HEC-RAS hydraulic model output indicates that the St. Croix River between St. Croix Falls, Wisconsin, and Stillwater, Minnesota, may support successful spawning of bighead carp for a range of discharge and water temperature. Across all simulated steady and unsteady discharges (283 to 991 m^3^/s), all spawning times using the unsteady simulations (Figs 2 and 4A; Table 1), and all water temperatures (22°C to 28°C; Fig 4A, Table 1), the percentage of spawned eggs that were predicted to hatch within the study reach had a mean of 49 percent and ranged from 1.4 to 91 percent. However, FluEgg simulations that used field-derived hydraulics collected primarily within the main channel for the same range of discharge predicted essentially no egg hatching within the study reach, regardless of the water temperature (Fig 4A; Table 2). Despite the conflicting results, there is sufficient evidence to conclude that successful spawning and hatching of bighead carp eggs is possible within the study reach. However, results from all 56 FluEgg simulations also predict that it is highly unlikely that bighead carp larvae could reach the gas bladder inflation stage within the study reach. Although this study focused primarily on bighead carp, these results could be generally applicable to silver and grass carp as well due to the similarities in egg characteristics and spawning requirements among species [26].

Notably, the results of FluEgg simulations driven with field data (simulations 1–11) are in stark contrast to the results from simulations driven by the 1D hydraulic model output (simulations 12–56). The reasons for this divergence appear to lie in the mean hydraulic conditions that each parameterization technique produces, and which are subsequently used as FluEgg inputs. In the case of the field-derived ADCP hydraulic measurements (i.e., flow depth, velocity, and shear velocity), logistical and safety concerns during high-flow, boat-based sampling precluded the collection of truly representative cross-sectional data that spanned the full suite of anabranches in the more braided reaches of the St. Croix River (Fig 1). As a result, ADCP sampling was completed primarily in the main channel, which could be safely sampled, but which also contained the highest flow velocities and the greatest flow depths, producing the highest shear stresses. Many transects did not capture the presumably shallower depths and slower flow velocities present in anabranches and backwater areas along the river that were inundated during the June 19–22, 2018 flows, and the resulting ADCP-derived inputs to FluEgg are most certainly biased toward greater depths and faster velocities. This bias propagates into the results of FluEgg simulations, manifesting as rapid transport of eggs downstream and complete export of any eggs from the study reach prior to hatching (Fig 4).

In contrast, 1D HEC-RAS modeling uses geometry derived from both measured cross sections and airborne lidar data across the entire floodplain; thus, each cross section generally describes the full suite of channels within that section. One-dimensional hydraulic models are not the optimal technique for simulating braided channels due to the presence of lateral gradients in shear stress and secondary circulation [52, 53]. Despite this, 1D models also often represent the best achievable hydraulic modeling scheme for a particular location, given the much greater topographic and bathymetric input data required to parameterize higher-order hydraulics of 2- and 3D models [53]; this was the case for the simulations completed as part of this study. The results of these simulations take into account the additional cross-sectional area and divided flow attributed to anabranches in braided reaches resulting in lower mean velocities and shallower mean depths for the cross section (Fig 6). The discrepancy between ADCP-derived hydraulics and HEC-RAS simulations is especially apparent in more braided reaches of the St. Croix study reach (Figs 1 and 6), where divergence between flow depths and velocities increases, with HEC-RAS producing markedly lower mean depth and mean velocity estimates for a cross section than were observed in thalweg-centric field surveys (e.g., transects 4–5 and 15–16 in Fig 1). Because HEC-RAS simulations result in reduced flow depths and velocities, a greater proportion of spawned eggs are predicted to remain within the study reach at hatching time, leading to markedly increased hatch probabilities for hydraulic model-driven FluEgg simulations (Fig 4; Table 2).

### 5.2. Controls on longitudinal distribution of eggs

Longitudinal egg positions at both hatching time and time to gas bladder inflation diverge substantially between FluEgg simulations parameterized with either field-based ADCP hydraulic data or the hydraulic outputs of HEC-RAS modeling (Fig 4B). These results are similar to the analysis of overall hatching success rates (Section 5.1). Egg plume longitudinal distributions reflect the effects of discharge, which modulates flow depth and velocity, and the temperature-dependent development time. In short, higher discharges and lower temperatures result in eggs that are transported farther downstream by hatching time, as a result of greater flow velocities and slower egg development. The opposite is true for warmer water temperatures and lower discharges, which ultimately lead to eggs hatching farther upstream.

In general, the entire egg plume was predicted to be located downstream from Stillwater, Minnesota, at egg hatching time for all temperatures simulated using FluEgg which were parameterized with field data. In contrast, 38 of the 45 HEC-RAS-driven FluEgg simulations predicted that at least 1 percent of the egg plume would be located within the study reach at hatching time and 16 steady-state simulations predicted the entire egg plume to be within the study reach at the time of hatching (Table 2). All 12 unsteady simulations resulted in the transport of eggs past Stillwater, Minnesota, and only simulation 56 exported fewer than 50% of the spawned eggs. Again, we hypothesize that the thalweg-centric nature of the field surveys during June 2018 resulted in estimates of flow depth and velocity that were higher than mean hydraulic conditions across the full cross section (including the numerous anabranches and backwater areas found throughout this reach). In turn this leads to more rapid egg advection downstream and in general, longer transport distances of the egg mass as simulated using FluEgg.

### 5.3. Implications for invasive carp monitoring and river management

The results of FluEgg simulations driven by field data differ appreciably from those derived using 1D steady and unsteady HEC-RAS hydraulics. Therefore, it is reasonable to ask whether one modeling approach provides a more ‘correct’ prediction, which in this case we use to indicate a particular model’s ability to accurately describe the bighead carp egg location and hatching success that would be observed in the field. We surmise that neither approach produces a completely accurate, nor field-realistic result, because both the ADCP- and HEC-RAS-driven FluEgg simulations represent simplified versions of the true hydraulics within the study reach. In the case of the ADCP-driven FluEgg simulations, sampling primarily within the channel thalweg likely produces estimates of egg dispersion that are biased toward more rapid transport, resulting in eggs being exported from the study reach and correspondingly low hatch probabilities between St. Croix Falls and Stillwater.

In the case of the HEC-RAS driven simulations, the use of 1D modeling and coarsely-spaced cross-sectional data results in highly simplified hydraulics for drift modeling, which necessarily fails to fully capture the complex, multi-dimensional hydrodynamics found on this and other multi-thread reaches [52]. Furthermore, the 1D modeling approach used here produces one mean value for flow depth, velocity, and shear stress at each cross section that effectively averages the multiple channels traversing that cross section in braided reaches. Compounding this simplification is the fact that 1D hydraulic simulations cannot model additional aspects of the flow field that may keep eggs in suspension, maintain their position in certain areas of the channel, or substantially alter dispersion of the egg plume. Although the unsteady HEC-RAS modeling approach better describes the time-variant hydraulics present during spawning events, the hydraulics are still spatially limited by the 1D modeling approach. Multi-dimensional, unsteady models can improve FluEgg predictions; however, such models generally require substantially more time and effort to develop and run. Furthermore, as of August 2021, FluEgg does not support 2D or 3D hydraulic inputs.

Based on the simplifications made by each approach for parameterizing FluEgg, we conclude that the ADCP and HEC-RAS cases likely represent end-member scenarios and that the true potential for successful suspension and hatching of bighead carp eggs in the St. Croix River upstream from Stillwater, Minnesota, (a critical first step in recruitment) lies somewhere between the predictions of the two approaches. That is, actual egg transport distances are likely not as large as the ADCP parameterization of FluEgg would predict yet would be longer than the HEC-RAS-driven scheme produced. The same is true for egg hatch probabilities, whereby the true successful hatching rate would be found between the estimates of the ADCP-driven FluEgg approach and the upper-bound estimates of the HEC-RAS steady-state method. The unsteady HEC-RAS approach produces results that fall between the FluEgg predictions from the ADCP and steady-state HEC-RAS methods and are likely the most accurate predictions from this study with respect to true hatching rates and transport distances. Among the unsteady model runs, we hypothesize that simulations 51–56, run at the hydrograph peak and on the descending limb of the hydrograph while discharge remained elevated, depict likely field conditions for successful bighead carp spawning [51]. However, we simulated one event at one water temperature, and there are an infinite number of spawning scenarios that would produce different results. In addition, given the plasticity in spawning traits of bigheaded carps [54], successful spawning during relatively low and stable flows (such as simulations 12–22) may be more common than expected. While the ADCP hydraulics necessarily over-predict egg transport rate, HEC-RAS hydraulics result in the averaging-out of discrete areas of high velocity because of the 1D nature of those simulations, thus resulting in what is likely an under-prediction of downstream egg transport rate. Nevertheless, the HEC-RAS simulations *do* account for the retention of water (and eggs) caused by the braided nature of the St. Croix River, and thus likely predict the downstream dispersion of eggs with greater fidelity than the ADCP-driven simulation, especially for the unsteady simulations. While the time-varying discharge and geomorphically complex nature of the St. Croix River necessitated unsteady modeling, there are scenarios where steady-state or field-derived hydraulics would be preferable for parameterizing biophysical drift models such as FluEgg. This is particularly true in single-thread rivers where field surveyed cross-sections can rapidly capture hydraulic characteristics across the channel and eliminate the need for more time-consuming continuous topographic and bathymetric coverage, or in systems where discharge is relatively invariant over the egg and larvae drift period.

The next logical step in parameterizing FluEgg would be to conduct a two-dimensional hydrodynamic modeling exercise, whereby the lateral component of flow could be derived, along with spatially continuous outputs of flow depth and velocity throughout the study reach, as opposed to the single values at cross sections spaced approximately every 0.3 km used here. Of course, FluEgg would need to be updated to accept 2D hydraulics and the parameterization and validation of a two-dimensional modeling approach would require more detailed data on channel depth and roughness characteristics than were available for our purposes in this study, and thus may need to be conducted over a shorter subreach of the St. Croix River. At the same time, such a targeted analysis would allow us to estimate the overall predictive capacity of a simple 1D hydraulic modeling approach by comparing the outputs of the two schemes.

Apart from the simplified nature of the hydraulic parameters used to drive FluEgg simulations, we also note that the use of a single constant water temperature in all model runs likely oversimplifies the true nature of water conditions encountered by invasive carp eggs in this reach of the St. Croix River. While the modeled temperature range (18–28°C) is representative of the conditions sampled during the June 2018 field effort, the heterogeneous nature of the river planform and flow depth throughout the study reach likely gives rise to patches and layers of water that vary appreciably in temperature over short distances and diurnally. The cumulative effect of encountering these areas of variable temperature ultimately determine egg hatching time and time to gas bladder inflation. Estimating temperature at discrete locations along the study reach was beyond the scope of this study and may have also proved impossible given the large areal extent of unsampled areas in the field data. However, we acknowledge that incorporating spatially and temporally varying water temperature has the potential to affect biological development; to our knowledge no such developmental studies have been completed, and this represents a knowledge gap in invasive carp research that could be addressed. Variable water temperature could drive shifts in egg hatching time and change the longitudinal distribution of the egg plume at this critical point in the recruitment process. While necessary for this study, the constant temperatures used in modeling represent a potentially consequential simplification in our work.

Nevertheless, the results of this study may provide valuable guidance for natural resource managers. At the very least, this study indicates that the lower St. Croix River may be treated as an invasive carp spawning reach and monitoring for future spawning activity would be warranted. Furthermore, this study can be used to guide field sampling efforts aimed at the detection and removal of invasive carp eggs and larvae. In the cases where our simulations predicted that portions of the egg plume would be located within the study reach at the time of hatching, the mean position of the egg mass could be used as initial guidance for detecting the presence of invasive carp, or areas to implement control efforts.

Finally, the overall potential for recruitment is particularly concerning for invasive carp management. While recruitment is dependent on a number of factors that are not simulated in FluEgg, the results of this study can inform recruitment risk assessments in the St. Croix River. Results from FluEgg simulations provide quantitative estimates of egg suspension until hatching, the first step in invasive carp recruitment. While FluEgg estimates represent an idealized scenario due to lack of a mortality model that includes predation and other factors, such estimates can be informative and be used as a screening tool for identification of rivers that meet the hydraulic requirements of these pelagic-spawning invasive carp. Furthermore, FluEgg modeling can help assess if invasive carp larvae reach the gas bladder inflation stage, the stage at which they develop the ability to swim horizontally and seek out nursery habitat, in river reaches where nursery habitat is present and accessible—the next step in recruitment. This study indicates that within the study reach, bighead carp eggs may hatch in substantial proportions under the right conditions, but the larvae will not reach the gas bladder inflation stage upstream from Stillwater, Minnesota. This finding is important because the study reach contains ideal nursery habitat, and such habitat is much sparser downstream from Stillwater. The lack of nursery habitat in Lake St. Croix may reduce overall recruitment potential for the St. Croix River.

Results of presented model simulations indicate that hatched bighead carp larvae would not reach the gas bladder inflation stage prior to entering Lake St. Croix downstream from the study reach. However, invasive carp recruitment could still threaten St. Croix River aquatic ecosystems. Significant increases in summer peak flows from rainfall events, number of high flow days, summer base flows, annual mean precipitation, number of intense rainfall events, and days with precipitation for rivers in Minnesota are expected based on trend analysis [55]. Furthermore, stream water temperatures in Minnesota are highly correlated with air temperatures [56], and flow models have been combined with future climate scenarios to illustrate increases in stream water temperatures corresponding to increases in air temperatures for a watershed in north-central Wisconsin [57]. Finally, trend analysis of long-term streamflow records for the St. Croix River at St. Croix Falls demonstrated significant increases in streamflows between 1914 and 2001 [58]. Therefore, increased water temperatures and more rainfall-driven summer peak flows are likely in the St. Croix River and could increase the probability that larval bighead carp can hatch and reach the gas bladder inflation stage near suitable nursery habitats in the lower St. Croix River. Climate model outputs could be combined with the underlying HEC-RAS model and FluEgg to examine how changes in water temperature and hydraulics affect hatching and development of bighead carp larvae, but these analyses are beyond the scope of this study.

## 6. Conclusions

Since their introduction to the United States in the 1960s and 1970s, invasive carp have steadily spread throughout the Mississippi River Basin, where they directly and indirectly compete with native fish and affect water quality. Because of their detrimental effects on native aquatic food webs, the potential spread and colonization of these carp are particularly concerning for scientists and resource managers developing invasive carp prevention strategies that also allow for sustainable management of native species and aquatic communities.

Here we used the FluEgg invasive carp drift model to predict bighead carp egg and larval distributions and hatching success rates along a 47.8-km reach of the St. Croix River between St. Croix Falls, Wisconsin, and Stillwater, Minnesota. FluEgg requires user-input data describing channel hydraulics, which we obtained using three different approaches. In the first approach, we used information on flow depth and velocity obtained via ADCP data collection during a high-flow event in June 2018; these data primarily covered faster-flowing areas of the main channel and thalweg that could be safely and rapidly surveyed from a moving boat. In the second and third approaches, we used the outputs of steady and unsteady 1D hydraulic models, respectively, that were built using simplified channel geometry, but which encompassed the full range of anabranches found in the study reach.

We found that the approaches produced considerably divergent predictions of invasive carp spawning suitability. In general, ADCP-parameterized FluEgg simulations predicted that eggs would be transported out of the study reach and into Lake St. Croix before hatching, increasing the potential for egg mortality resulting from settling (Fig 4). However, the HEC-RAS parameterized simulations predicted retention of substantial proportions of eggs within the study reach, with correspondingly higher rates of egg hatching upstream from Lake St. Croix. In general, unsteady modeling produced lower in-river hatching rates than steady-state modeling (Fig 4). These divergent conclusions highlight the importance of user-defined input data into invasive carp drift models, particularly for hydraulically and geomorphically heterogeneous rivers, and we conclude that they represent end-member scenarios that describe the reasonable bounds of egg dispersion along the St. Croix River. Our results indicate that the unsteady modelling approach provides the most accurate predictions of the three methods used in this study.

Overall, the results of this study indicate that the lower St. Croix River can support spawning and reproduction of invasive carp for a range of discharge and water temperatures. Two-dimensional hydraulic modeling, which can more accurately represent the spatial variability of the flow field and predict downstream egg and larvae transport, could help to converge on a high-fidelity prediction of the suitability of this reach for invasive carp reproduction despite requiring a higher overhead of input boundary data and computing power. Finally, our results have implications for both parameterizing invasive carp drift models and for helping to guide natural resource managers in monitoring and control efforts.
